# Supplementary material for: A Likelihood Approach for Real-Time Calibration of Stochastic Compartmental Epidemic Models
Source: PLoS Comput Biol. 2017 Jan 17;13(1):e1005257. doi: 10.1371/journal.pcbi.1005257 (PMC5240920; doi:10.1371/journal.pcbi.1005257)
Supplement: S1 File — (TAR.GZ) [file pcbi.1005257.s014.tar.gz › HSPH_Online-SI-Revision/EnKF07/n1-severe/n1-severe_EnKF2_table8.pdf]

1	newly 3 weeks spec	10.	13.73	{0., 17.}	133.5	0.680739	newly 3 weeks cumul	63.	94.46	{19., 103.}	135.27	0.385609	newly 5 weeks spec	19.	6.82	{0., 9.}	102.105	1.42905	newly 5 weeks cumul	93.	110.65	{19., 129.}	124.204	0.476112	newly 46 weeks cumul	179.	132.38	{20., 378.}	107.955	0.667664
2	newly 3 weeks spec	13.	6.85	{0., 13.}	105.308	1.36404	newly 3 weeks cumul	51.	97.75	{13., 192.}	133.118	0.348092	newly 5 weeks spec	4.	1.56	{0., 6.}	103.	1.33243	newly 5 weeks cumul	64.	101.98	{13., 192.}	111.	0.351957	newly 48 weeks cumul	85.	104.61	{13., 192.}	93.6353	0.39367
3	newly 3 weeks spec	16.	18.19	{0., 31.}	130.813	0.86125	newly 3 weeks cumul	56.	106.51	{8., 293.}	149.161	0.430117	newly 5 weeks spec	18.	6.78	{0., 12.}	104.333	1.5093	newly 5 weeks cumul	91.	124.26	{8., 371.}	123.648	0.517264	newly 47 weeks cumul	131.	135.04	{8., 379.}	110.321	0.609953
4	newly 3 weeks spec	8.	12.71	{0., 34.}	177.375	0.937756	newly 3 weeks cumul	27.	73.92	{7., 167.}	217.852	0.402066	newly 5 weeks spec	8.	5.08	{0., 15.}	115.	1.38158	newly 5 weeks cumul	40.	86.83	{7., 208.}	181.725	0.447676	newly 50 weeks cumul	56.	101.53	{9., 295.}	163.518	0.517322
5	newly 3 weeks spec	13.	11.4	{0., 21.}	128.923	1.2667	newly 3 weeks cumul	49.	168.05	{9., 452.}	286.02	0.500333	newly 5 weeks spec	5.	4.32	{0., 4.}	155.2	1.40808	newly 5 weeks cumul	61.	178.72	{9., 587.}	246.557	0.515235	newly 49 weeks cumul	65.	188.2	{9., 587.}	245.292	0.52532
6	newly 3 weeks spec	15.	5.04	{0., 10.}	88.2667	1.1553	newly 3 weeks cumul	74.	43.5	{8., 55.}	85.9459	0.600279	newly 5 weeks spec	23.	2.92	{0., 4.}	98.	1.94242	newly 5 weeks cumul	114.	49.84	{8., 55.}	88.1404	0.739034	newly 46 weeks cumul	183.	70.24	{8., 75.}	95.9563	0.904972
7	newly 3 weeks spec	8.	25.02	{0., 67.}	290.	0.746695	newly 3 weeks cumul	25.	149.84	{13., 480.}	520.88	0.483188	newly 5 weeks spec	2.	9.21	{0., 27.}	450.5	0.855477	newly 5 weeks cumul	27.	173.46	{14., 706.}	563.481	0.511684	newly 46 weeks cumul	44.	197.93	{16., 818.}	385.25	0.460029
8	newly 3 weeks spec	0.1	10.26	{0., 18.}	10222.	1.2388	newly 3 weeks cumul	0.1	126.79	{8., 336.}	126692.	2.62178	newly 5 weeks spec	0.1	2.72	{0., 6.}	2748.	0.564361	newly 5 weeks cumul	0.1	133.99	{9., 484.}	133892.	2.65933	newly 50 weeks cumul	0.1	140.43	{9., 579.}	140332.	2.68891
9	newly 3 weeks spec	22.	19.83	{0., 32.}	110.5	0.881211	newly 3 weeks cumul	56.	112.65	{13., 129.}	152.946	0.359535	newly 5 weeks spec	13.	10.08	{0., 16.}	114.615	1.18501	newly 5 weeks cumul	88.	136.46	{13., 203.}	126.273	0.408527	newly 46 weeks cumul	139.	172.57	{13., 333.}	117.719	0.513634
10	newly 3 weeks spec	10.	12.3	{0., 20.}	144.6	1.04018	newly 3 weeks cumul	37.	101.78	{9., 113.}	212.595	0.398155	newly 5 weeks spec	2.	5.08	{0., 10.}	281.	0.987165	newly 5 weeks cumul	45.	114.18	{9., 135.}	193.2	0.386217	newly 46 weeks cumul	50.	125.44	{9., 175.}	192.16	0.394969
11	newly 3 weeks spec	17.	6.27	{0., 17.}	90.5294	1.41928	newly 3 weeks cumul	63.	45.53	{3., 82.}	88.8413	0.651276	newly 5 weeks spec	10.	2.16	{0., 5.}	96.	1.62574	newly 5 weeks cumul	90.	51.12	{3., 96.}	87.3556	0.758198	newly 43 weeks cumul	187.	56.47	{3., 113.}	84.9893	1.01198
12	newly 3 weeks spec	0.1	14.7	{0., 13.}	14644.	1.35087	newly 3 weeks cumul	10.	55.78	{4., 68.}	477.8	0.512978	newly 5 weeks spec	0.1	6.89	{0., 7.}	6882.	0.845126	newly 5 weeks cumul	10.	73.11	{5., 72.}	648.3	0.572236	newly 51 weeks cumul	10.	84.34	{5., 121.}	760.	0.621906
13	newly 3 weeks spec	9.	4.16	{0., 6.}	113.778	1.47959	newly 3 weeks cumul	45.	75.93	{5., 211.}	157.311	0.532903	newly 5 weeks spec	9.	1.7	{0., 7.}	106.889	1.78274	newly 5 weeks cumul	69.	80.2	{5., 241.}	128.551	0.643266	newly 46 weeks cumul	95.	86.09	{5., 241.}	119.168	0.745972
14	newly 3 weeks spec	8.	19.31	{0., 27.}	194.125	0.482722	newly 3 weeks cumul	35.	122.12	{12., 231.}	274.571	0.370761	newly 5 weeks spec	7.	9.94	{0., 12.}	150.	0.755924	newly 5 weeks cumul	50.	145.46	{15., 260.}	222.04	0.346683	newly 51 weeks cumul	59.	172.4	{20., 404.}	225.39	0.360166
15	newly 3 weeks spec	1.	11.48	{0., 38.}	1112.	0.986273	newly 3 weeks cumul	11.	132.56	{10., 380.}	1112.	0.784358	newly 5 weeks spec	1.	3.36	{0., 9.}	326.	0.808546	newly 5 weeks cumul	12.	142.16	{11., 415.}	1091.83	0.793559	newly 45 weeks cumul	13.	147.48	{13., 425.}	1041.08	0.790408
16	newly 3 weeks spec	17.	14.56	{0., 33.}	116.706	1.06103	newly 3 weeks cumul	40.	99.61	{8., 129.}	188.475	0.360102	newly 5 weeks spec	8.	6.95	{0., 10.}	137.375	1.26171	newly 5 weeks cumul	60.	115.79	{9., 196.}	149.383	0.3762	newly 51 weeks cumul	89.	138.58	{11., 372.}	137.483	0.458307
17	newly 3 weeks spec	3.	23.63	{1., 77.}	720.333	0.703405	newly 3 weeks cumul	13.	185.89	{13., 627.}	1340.08	0.779075	newly 5 weeks spec	2.	8.9	{0., 19.}	434.	0.850208	newly 5 weeks cumul	18.	209.32	{13., 628.}	1074.89	0.728785	newly 51 weeks cumul	18.	233.06	{16., 880.}	1205.22	0.780812
18	newly 3 weeks spec	24.	14.76	{2., 24.}	87.25	0.684419	newly 3 weeks cumul	73.	117.54	{17., 163.}	140.329	0.417554	newly 5 weeks spec	26.	5.4	{0., 13.}	83.9231	1.25817	newly 5 weeks cumul	128.	131.28	{18., 220.}	111.766	0.537536	newly 51 weeks cumul	297.	148.98	{19., 299.}	91.6229	0.762767
19	newly 3 weeks spec	13.	7.14	{0., 3.}	133.538	1.58593	newly 3 weeks cumul	52.	44.36	{1., 30.}	127.5	0.793304	newly 5 weeks spec	8.	1.15	{0., 0.}	104.375	1.78038	newly 5 weeks cumul	69.	48.67	{1., 31.}	123.754	0.898821	newly 44 weeks cumul	105.	49.4	{1., 31.}	113.238	1.06464
20	newly 3 weeks spec	5.	8.56	{0., 20.}	171.2	0.9644	newly 3 weeks cumul	13.	159.99	{10., 733.}	1140.23	0.754473	newly 5 weeks spec	1.	4.22	{0., 13.}	454.	0.96944	newly 5 weeks cumul	19.	169.98	{10., 734.}	809.684	0.675057	newly 44 weeks cumul	36.	181.76	{10., 734.}	430.667	0.540745
21	newly 3 weeks spec	15.	7.69	{0., 26.}	106.333	1.35593	newly 3 weeks cumul	44.	71.08	{5., 149.}	163.5	0.56367	newly 5 weeks spec	7.	2.73	{0., 5.}	109.571	1.5184	newly 5 weeks cumul	62.	78.07	{5., 280.}	142.306	0.654947	newly 51 weeks cumul	100.	81.49	{5., 280.}	115.75	0.793279
22	newly 3 weeks spec	1.	5.35	{0., 14.}	497.	0.778832	newly 3 weeks cumul	13.	128.13	{12., 465.}	896.538	0.70546	newly 5 weeks spec	0.1	1.2	{0., 4.}	1230.	0.500928	newly 5 weeks cumul	13.	131.68	{14., 465.}	923.231	0.731293	newly 45 weeks cumul	13.	134.26	{14., 465.}	942.769	0.747432
23	newly 3 weeks spec	12.	7.42	{0., 10.}	111.667	1.26025	newly 3 weeks cumul	38.	100.82	{7., 401.}	241.895	0.481787	newly 5 weeks spec	6.	5.21	{0., 4.}	150.167	1.30207	newly 5 weeks cumul	50.	113.24	{9., 405.}	213.72	0.527497	newly 47 weeks cumul	82.	125.28	{9., 440.}	161.634	0.629303
24	newly 3 weeks spec	7.	8.91	{0., 9.}	183.	1.2452	newly 3 weeks cumul	21.	62.9	{3., 61.}	265.619	0.425082	newly 5 weeks spec	2.	2.3	{0., 2.}	186.	1.18166	newly 5 weeks cumul	24.	69.39	{3., 112.}	263.792	0.460884	newly 44 weeks cumul	30.	74.59	{3., 133.}	238.967	0.521587
25	newly 3 weeks spec	3.	14.66	{0., 14.}	446.667	0.583532	newly 3 weeks cumul	19.	61.24	{3., 70.}	265.053	0.454113	newly 5 weeks spec	2.	4.55	{0., 8.}	249.5	0.886314	newly 5 weeks cumul	27.	73.87	{4., 83.}	222.778	0.433946	newly 42 weeks cumul	28.	80.44	{4., 83.}	235.357	0.440981
26	newly 3 weeks spec	6.	15.55	{0., 30.}	274.5	1.00044	newly 3 weeks cumul	26.	112.5	{10., 284.}	356.154	0.461106	newly 5 weeks spec	13.	6.27	{0., 6.}	121.	1.64599	newly 5 weeks cumul	53.	128.37	{12., 284.}	190.623	0.377882	newly 49 weeks cumul	100.	146.72	{16., 498.}	144.46	0.534788
27	newly 3 weeks spec	0.1	10.25	{0., 27.}	10210.	1.18969	newly 3 weeks cumul	0.1	78.65	{9., 211.}	78550.	2.55488	newly 5 weeks spec	0.1	3.84	{0., 7.}	3868.	0.562809	newly 5 weeks cumul	0.1	88.65	{11., 237.}	88550.	2.59437	newly 48 weeks cumul	0.1	93.85	{11., 260.}	93750.	2.60498
28	newly 3 weeks spec	18.	17.58	{0., 15.}	124.444	0.862137	newly 3 weeks cumul	61.	75.73	{9., 71.}	110.934	0.442621	newly 5 weeks spec	6.	9.55	{0., 15.}	179.5	0.923852	newly 5 weeks cumul	78.	97.88	{10., 78.}	117.821	0.47736	newly 51 weeks cumul	104.	121.29	{11., 175.}	119.029	0.542118
29	newly 3 weeks spec	6.	23.25	{0., 96.}	390.5	0.955342	newly 3 weeks cumul	22.	103.09	{5., 220.}	409.864	0.511069	newly 5 weeks spec	6.	10.58	{0., 21.}	219.	1.21169	newly 5 weeks cumul	37.	128.72	{5., 424.}	305.676	0.498069	newly 44 weeks cumul	89.	152.53	{7., 498.}	180.64	0.680477
30	newly 3 weeks spec	5.	16.98	{0., 47.}	321.2	0.793926	newly 3 weeks cumul	19.	106.79	{9., 341.}	486.895	0.483845	newly 5 weeks spec	1.	6.9	{0., 8.}	698.	0.825381	newly 5 weeks cumul	23.	124.35	{9., 469.}	468.217	0.465107	newly 45 weeks cumul	36.	133.87	{10., 473.}	314.194	0.418096
31	newly 3 weeks spec	5.	11.85	{0., 16.}	231.4	0.782617	newly 3 weeks cumul	36.	171.42	{21., 668.}	395.444	0.398691	newly 5 weeks spec	7.	2.52	{0., 4.}	102.857	1.42186	newly 5 weeks cumul	48.	179.08	{21., 685.}	302.667	0.367883	newly 46 weeks cumul	144.	183.71	{21., 696.}	132.965	0.548691
32	newly 3 weeks spec	16.	10.27	{0., 23.}	122.313	1.40135	newly 3 weeks cumul	65.	54.08	{4., 139.}	109.262	0.726901	newly 5 weeks spec	15.	3.79	{0., 6.}	102.6	1.8451	newly 5 weeks cumul	94.	64.15	{4., 180.}	105.862	0.838648	newly 48 weeks cumul	156.	72.2	{4., 210.}	97.5	1.00348
33	newly 3 weeks spec	6.	15.78	{0., 51.}	260.	0.859123	newly 3 weeks cumul	27.	91.73	{7., 172.}	280.037	0.42675	newly 5 weeks spec	2.	5.18	{0., 12.}	280.	0.932673	newly 5 weeks cumul	33.	106.75	{7., 288.}	269.788	0.445688	newly 51 weeks cumul	47.	120.66	{7., 353.}	221.745	0.488023
34	newly 3 weeks spec	10.	6.32	{0., 6.}	124.8	1.39767	newly 3 weeks cumul	29.	78.7	{4., 45.}	257.31	0.473418	newly 5 weeks spec	9.	0.75	{0., 1.}	97.8889	1.7647	newly 5 weeks cumul	48.	81.84	{5., 45.}	188.292	0.616556	newly 43 weeks cumul	102.	82.9	{5., 55.}	131.49	0.87628
35	newly 3 weeks spec	0.1	10.8	{0., 16.}	10782.	1.11117	newly 3 weeks cumul	3.	123.91	{9., 255.}	4035.67	1.24821	newly 5 weeks spec	0.1	5.21	{0., 6.}	5240.	0.573635	newly 5 weeks cumul	3.	136.09	{9., 299.}	4441.67	1.28604	newly 51 weeks cumul	3.	154.99	{9., 692.}	5071.67	1.3117
36	newly 3 weeks spec	0.1	10.28	{0., 20.}	10224.	1.33901	newly 3 weeks cumul	0.1	57.49	{9., 72.}	57390.	2.52226	newly 5 weeks spec	0.1	4.83	{0., 11.}	4838.	0.791687	newly 5 weeks cumul	0.1	69.01	{10., 95.}	68910.	2.57558	newly 52 weeks cumul	0.1	83.5	{10., 137.}	83400.	2.62048
37	newly 3 weeks spec	18.	14.99	{1., 26.}	88.2778	0.605449	newly 3 weeks cumul	45.	53.95	{8., 91.}	76.3778	0.343542	newly 5 weeks spec	10.	10.47	{0., 25.}	114.9	0.801847	newly 5 weeks cumul	67.	77.45	{11., 124.}	83.1493	0.379453	newly 42 weeks cumul	100.	124.99	{14., 292.}	108.03	0.476984
38	newly 3 weeks spec	3.	11.96	{0., 33.}	382.667	0.843015	newly 3 weeks cumul	8.	100.13	{8., 98.}	1161.63	0.70885	newly 5 weeks spec	0.1	5.93	{0., 7.}	5922.	0.864294	newly 5 weeks cumul	8.	114.05	{9., 180.}	1334.38	0.758479	newly 50 weeks cumul	8.	141.6	{9., 456.}	1677.	0.807677
39	newly 3 weeks spec	6.	12.85	{0., 27.}	250.167	1.13583	newly 3 weeks cumul	26.	89.6	{2., 238.}	320.154	0.574397	newly 5 weeks spec	6.	4.73	{0., 11.}	143.833	1.46618	newly 5 weeks cumul	37.	101.25	{3., 388.}	265.108	0.629006	newly 46 weeks cumul	74.	121.15	{3., 505.}	187.77	0.821008
40	newly 3 weeks spec	9.	12.42	{1., 26.}	129.556	0.564685	newly 3 weeks cumul	26.	91.75	{14., 190.}	272.731	0.405099	newly 5 weeks spec	4.	3.7	{0., 7.}	118.	0.871339	newly 5 weeks cumul	37.	102.15	{14., 224.}	199.757	0.340912	newly 52 weeks cumul	38.	108.93	{14., 263.}	209.132	0.348347
41	newly 3 weeks spec	0.1	7.4	{0., 15.}	7410.	0.871829	newly 3 weeks cumul	9.	122.62	{12., 325.}	1266.22	0.813767	newly 5 weeks spec	0.1	2.58	{0., 7.}	2630.	0.415559	newly 5 weeks cumul	9.	129.24	{13., 385.}	1339.78	0.83737	newly 49 weeks cumul	9.	138.85	{13., 397.}	1446.56	0.860787
42	newly 3 weeks spec	13.	13.83	{0., 49.}	118.385	0.801039	newly 3 weeks cumul	42.	140.93	{20., 330.}	267.024	0.330117	newly 5 weeks spec	12.	3.33	{0., 9.}	91.5833	1.45397	newly 5 weeks cumul	62.	151.19	{23., 454.}	200.21	0.370628	newly 49 weeks cumul	154.	155.59	{23., 503.}	116.851	0.593683
43	newly 3 weeks spec	2.	12.45	{0., 17.}	577.5	0.673529	newly 3 weeks cumul	22.	76.3	{9., 98.}	273.364	0.378785	newly 5 weeks spec	4.	3.31	{0., 7.}	123.75	1.05676	newly 5 weeks cumul	31.	85.57	{10., 115.}	211.645	0.347576	newly 42 weeks cumul	85.	91.51	{10., 139.}	111.659	0.529013
44	newly 3 weeks spec	4.	19.88	{0., 33.}	435.5	0.663962	newly 3 weeks cumul	11.	114.23	{17., 220.}	942.273	0.815287	newly 5 weeks spec	3.	7.59	{0., 17.}	237.	0.806696	newly 5 weeks cumul	24.	133.93	{17., 252.}	469.042	0.586951	newly 50 weeks cumul	29.	151.79	{17., 366.}	436.862	0.569905
45	newly 3 weeks spec	11.	11.14	{0., 24.}	149.273	1.2443	newly 3 weeks cumul	31.	62.86	{6., 143.}	155.355	0.399856	newly 5 weeks spec	14.	5.59	{0., 5.}	115.214	1.77024	newly 5 weeks cumul	50.	76.37	{6., 158.}	136.94	0.493464	newly 47 weeks cumul	85.	90.19	{6., 164.}	121.047	0.638199
46	newly 3 weeks spec	18.	1.34	{0., 4.}	95.7778	1.96642	newly 3 weeks cumul	58.	70.71	{1., 66.}	126.259	0.648418	newly 5 weeks spec	11.	0.4	{0., 0.}	99.0909	1.94614	newly 5 weeks cumul	91.	71.86	{1., 66.}	109.648	0.799866	newly 46 weeks cumul	196.	72.21	{1., 66.}	95.4949	1.07465
47	newly 3 weeks spec	0.1	11.17	{0., 18.}	11128.	1.18592	newly 3 weeks cumul	0.1	89.32	{10., 218.}	89220.	2.5489	newly 5 weeks spec	0.1	2.51	{0., 6.}	2548.	0.479159	newly 5 weeks cumul	0.1	97.99	{10., 241.}	97890.	2.57645	newly 52 weeks cumul	0.1	100.92	{10., 241.}	100820.	2.58451
48	newly 3 weeks spec	5.	4.11	{0., 13.}	120.6	1.21128	newly 3 weeks cumul	29.	88.19	{5., 161.}	257.483	0.455738	newly 5 weeks spec	5.	1.42	{0., 3.}	102.4	1.43918	newly 5 weeks cumul	37.	91.76	{6., 178.}	212.757	0.476326	newly 46 weeks cumul	57.	96.54	{6., 194.}	157.544	0.545687
49	newly 3 weeks spec	13.	10.08	{0., 22.}	104.462	1.07218	newly 3 weeks cumul	31.	84.64	{6., 120.}	200.774	0.347857	newly 5 weeks spec	3.	4.82	{0., 14.}	180.667	1.0017	newly 5 weeks cumul	42.	96.05	{16., 151.}	164.738	0.332231	newly 50 weeks cumul	46.	110.35	{16., 217.}	175.239	0.349384
50	newly 3 weeks spec	11.	8.1	{0., 12.}	124.	1.31783	newly 3 weeks cumul	42.	49.9	{3., 100.}	122.952	0.625918	newly 5 weeks spec	10.	4.96	{0., 4.}	124.	1.6526	newly 5 weeks cumul	58.	61.56	{3., 137.}	122.621	0.709708	newly 48 weeks cumul	98.	75.91	{3., 195.}	113.235	0.867525
